# A Review of Bacterial Co-Infections in Farmed Catfish: Components, Diagnostics, and Treatment Directions

**DOI:** 10.3390/ani11113240

**Published:** 2021-11-12

**Authors:** Allison L. Wise, Benjamin R. LaFrentz, Anita M. Kelly, Lester H. Khoo, Tingbi Xu, Mark R. Liles, Timothy J. Bruce

**Affiliations:** 1 School of Fisheries, Aquaculture, and Aquatic Sciences, College of Agriculture, Auburn University, Auburn, AL 36829, USA; alw0144@auburn.edu (A.L.W.); amk0105@auburn.edu (A.M.K.); 2 Aquatic Animal Health Research Unit, United States Department of Agriculture, Agricultural Research Service, Auburn, AL 36832, USA; benjamin.lafrentz@usda.gov; 3 Thad Cochran National Warmwater Aquaculture Center, Mississippi State University, Stoneville, MS 38776, USA; khoo@cvm.msstate.edu; 4 Department of Biological Sciences, College of Sciences and Mathematics, Auburn University, Auburn, AL 36849, USA; tzx0017@auburn.edu (T.X.); lilesma@auburn.edu (M.R.L.)

**Keywords:** aquaculture, fish disease, co-infections, pathobiology, emerging diseases

## Abstract

**Simple Summary:**

Catfish aquaculture is a prominent agricultural sector for foodfish production in the Southern United States. Catfish producers often experience high-level mortality events due to bacterial pathogens. In many instances, co-infections caused by multiple bacterial fish pathogens are isolated during diagnostic cases. These bacterial–bacterial interactions may alter the infection dynamics, and many of these mechanisms and interactions remain unclear. Furthermore, these co-infections may complicate disease management plans and treatment strategies. The current review provides an overview of the prevalent bacterial pathogens in catfish culture and previously reported instances of co-infections in catfish and other production fish species.

**Abstract:**

Catfish production is a major aquaculture industry in the United States and is the largest sector of food fish production. As producers aim to optimize production yields, diseases caused by bacterial pathogens are responsible for high pond mortality rates and economic losses. The major bacterial pathogens responsible are *Edwardsiella ictaluri*, *Aeromonas* spp., and *Flavobacterium columnare*. Given the outdoor pond culture environments and ubiquitous nature of these aquatic pathogens, there have been many reports of co-infective bacterial infections within this aquaculture sector. Co-infections may be responsible for altering disease infection mechanics, increasing mortality rates, and creating difficulties for disease management plans. Furthermore, proper diagnoses of primary and secondary pathogens are essential in ensuring the correct treatment approaches for antimicrobials and chemical applications. A thorough understanding of the interactions and infectivity dynamics for these warm water bacterial pathogens will allow for the adoption of new prevention and control methods, particularly in vaccine development. This review aims to provide an overview of co-infective pathogens in catfish culture and present diagnostic case data from Mississippi and Alabama to define prevalence for these multiple-species infections better.

## 1. Catfish Culture in the Southern United States

Aquaculture provides safe and sustainable fish crops for consumers, supplying approximately 540,178 tons of total aquaculture production per year in the United States. Of this total, 65 percent of production originates from finfish alone. The most common production species include catfish, trout, salmon, and tilapia [1]. Nearly 300 million pounds of channel (*Ictalurus punctatus*) and hybrid catfish (*I. punctatus* ♀ × *I. furcatus* ♂, are produced annually in the United States, making it substantially larger than any other aquaculture industry valued at half a billion dollars per year. The production of freshwater catfish is dominated by the southeastern region of the United States, especially in Mississippi, Alabama, and Arkansas [2]. The Mississippi Delta’s economy relies heavily on the revenue generated through the catfish industry, and Mississippi’s catfish industry produced USD 223,972,000 in annual sales, with Alabama producing USD 98,763,000.

Catfish typically inhabit freshwater streams but are also found in brackish muddy waters, lakes, and ponds, which allows them to tolerate culture in earthen ponds, and the pond types often include levees and/or watershed designs [3]. Catfish culture systems differ between the two major catfish producing states in the U.S., with Alabama having mainly watershed ponds, while Mississippi has levee ponds or split-pond production systems with groundwater wells as the water source. The optimal growth temperature for channel catfish is around 29.4 °C, and environmental temperatures can dictate appetite. Increased temperatures, to an extent, lead to increased feed consumption, which in turn may influence growth mechanics. Initially, eggs are transferred to hatchery troughs following spawning. When the eggs hatch, the fry are transported and placed into production ponds, where they grow to fingerling sizes. Fingerlings are then subjected to grow-out and are harvested once a marketable weight (approximately 1½ lbs) is reached [4].

Though channel catfish are the most common catfish produced in the USA, the production of hybrid catfish has been increased by producers due to higher survival rates, crop yields, resistance to certain pathogens, and health benefits [5,6]. Hybrids are produced by artificially breeding female channel catfish with male blue catfish (*Ictalurus furcatus*). Female channel catfish are injected with hormones to induce ovulation, and sperm is removed from male blue catfish and used to fertilize eggs. Inconsistent egg quality and poor hatchery conditions increase the difficulty of hybrid production and hybrid fry production costs [5]. However, genetic advances have been made, resulting in the increased efficiency of hybrid fry production. Though hybrid catfish have presented traits of increased resistance to pathogens compared to channel catfish, diseases are still a concern for both [7]. Methods for controlling diseases have included medicated feed, improved water quality, vaccines, and genetic improvement [7]. For both channel and hybrid catfish, diseases are a primary concern for farmers, which has spurred an investigation into disease pathogens and co-infections to develop effective disease management and treatment practices. As such, an understanding of the primary disease mechanisms for common bacterial pathogens in catfish culture is essential to discern dynamics related to bacterial co-infections.

## 2. Bacterial Pathogens Commonly Observed in Catfish Culture

Due to the economic impact of the catfish industry in the southeastern United States, maintaining catfish health is the primary concern for most farmers. Overcrowding and elevated temperatures facilitate disease within the hatcheries and ponds, resulting in high mortality rates and profit declines due to mortalities and costs associated with treatment. The majority of disease-related deaths in the catfish industry originate from bacterial diseases, with the most prominent being *Edwardsiella ictaluri*, *Flavobacterium columnare*, and *Aeromonas hydrophila* in channel catfish; 78.1% of production operations and 42.1% of ponds experience outbreaks of enteric septicemia of catfish (ESC) and columnaris disease [8]; (Summary provided in Table 1). Each pathogen is responsible for causing substantial catfish mortalities and creating interruptions to production due to lost feeding time (growth) and mitigation with chemical or antibiotic treatment means (economic expenses). Extensive efforts from researchers have been devoted to developing vaccines and other methods to reduce the losses caused by bacterial infections.

Economic losses in the aquaculture industry due to disease have assumed that infections are due to a single pathogen. Though often unreported, co-infections amongst these pathogens may lead to increased mortality and result in more severe economic losses. However, the mechanics and prevalence of these bacterial co-infections remain poorly documented. In order to properly diagnose and investigate co-infective factors involving bacterial pathogens, it is important to comprehend disease etiology and the current treatment options available. Improvements in treatment methods against single pathogen infections have increased over the years, yet many treatment effects have not been tested in the context of mixed infections.

### 2.1. Edwardsiella ictaluri

*Edwardsiella ictaluri* is the causative pathogen of ESC, an often fatal disease in catfish. Approximately USD 60 million in losses [9] and up to 47 percent of catfish disease cases in MS [10] have been attributed to ESC annually. *E. ictaluri* enters through the gut and passes from the stomach into the intestine. Clinical signs of ESC include petechial hemorrhaging around the head or mouth, pectoral fins, and abdomen. White nodules in the liver, exophthalmia, distended abdomen due to ascites, and cranial ulcerations are also present [10]. Fish can be seen swimming in spiral motions and swimming at the surface due to systemic infection and inflammation of the brain. Definitive pathogen diagnosis includes isolating *E. ictaluri* from internal organs, such as the brain, posterior kidney, or spleen, on tryptic soy agar with sheep blood [10]. Environmental conditions can impact *E. ictaluri* infection, with outbreaks typically associated with prominent levels of stress due to handling and confinement and temperature. The optimal temperature range for outbreaks is often within the 22–28 °C range. Transmission occurs once an infected fish sheds the pathogen, thus allowing surrounding fish to ingest *E. ictaluri* [10]. Although all ages of catfish can experience ESC, channel catfish fingerlings are most susceptible to post-hatch losses due to disease typically arising from ESC [11]. ESC survivors possess immunity to the disease, rendering older catfish more resistant to reinfection. In addition to *E. ictaluri* infections in catfish, other related and pathogenic *Edwardsiella* spp. are may also be isolated from catfish ponds, including *E. tarda* [12] and *E. piscicida* [13], although *E. piscicida* is more prevalent than *E. tarda*.

The management strategy and treatment for ESC often call for feed medicated with antibiotics, including sulfadimethoxine–ormetoprim and florfenicol. Other methods include restricting feed to the catfish while temperatures are within ESC’s optimal growth range. However, this method also has some limitations as this management strategy can reduce the growth of catfish during production [11]. Recently, several investigations aimed to discern mechanisms of virulence for *E. ictaluri*. Abdelhamed et al. (2017) identified the TonB transducing system as a virulence factor intertwined with ESC pathogenesis [14]. The role of lipopolysaccharides (LPS) in *E. ictaluri* virulence was also investigated, with selected mutations related to LPS biosynthesis resulting in such modifications as altered biofilm formation abilities and motility [15]. Understanding these virulence factors is of great importance for understanding the associated pathogenesis of ESC, and recently [16] identified differences in plasmids and virulence factors in *E. ictaluri* isolates from various fish species. Thus, the infectivity of *E. ictaluri* remains a topic of research interest to more thoroughly define entry and host-pathogen interactions mechanisms.

The development and introduction of ESC vaccines have proven to decrease infection rates, but it does not eliminate infection threats. Typically, ESC vaccines were administered through immersion baths on catfish fry [11]. However, this method may not be the most effective as fry may not be immunocompetent. In addition, it was documented that immunoglobulin responses cannot appear in channel catfish until three to four weeks post-hatch [17]. Therefore, fingerling vaccination is likely the most effective method. For catfish aquaculture, there are limited opportunities to vaccinate fingerlings using immersion delivery. Therefore, effective oral vaccines are preferred.

Live *E. ictaluri* vaccines received considerable attention due to the lack of immune response elicited from inactivated vaccines. However, live vaccines can generate many immune responses, thus allowing higher protective abilities than the killed vaccines [18]. Therefore, a live-attenuated oral vaccine was developed to combat the pathogen. The vaccine strain *E. ictaluri* S97-773-340X2 was attenuated by passage on medium containing increasing concentrations of rifampicin, a method previously reported by Klesius and Shoemaker (1999) [19]. The effectiveness of the live-attenuated ESC vaccine was tested using laboratory and experimental pond trials [20], and the results demonstrated significant increases in fingerling survival against multiple *E. ictaluri* field isolates [21]. Commercial feed was coated with the 340X attenuated isolate and administered to fingerling catfish orally [22]. Increased feed consumption of vaccinated fingerlings was documented along with decreased mortality due to disease compared to nonvaccinated fish [20]. In addition to survival, antibody responses were also measured. Vaccinated fish presented with an 18-fold increase in anti-*E. ictaluri* antibody levels when compared to nonvaccinated fish [20]. Antibody production is correlated to protective abilities against certain pathogens [23]. The results of the oral live-attenuated ESC vaccine experiments and pond trials have demonstrated that this is an effective avenue to protect channel catfish against ESC [20].

In addition to vaccination, the use of hybrids has reduced the impact of *E. ictaluri* infections on the industry. Hybrid catfish demonstrated considerable resistance to ESC, thus promoting their use to farmers and researchers. Blue catfish were also shown to have a higher resistance to ESC. Therefore, analyzing the resistance of multiple catfish species and families [24] has become increasingly important, providing the possibility for farmers to raise hybrids by taking advantage of hybrids’ ability to have increased resistance to disease [9].

### 2.2. Virulent Aeromonas hydrophila

Historically, *A. hydrophila* was considered a secondary pathogen in fish production, with cases of motile Aeromonad Septicemia (MAS) typically observed in fish that are stressed due to adverse environmental conditions or infection by a primary pathogen [25]. MAS is also inclusive of other *Aeromonas* spp. that may also be present in catfish ponds. Fish with MAS can exhibit various signs, especially hemorrhaging and lesions that can progress to necrotic ulcers, and MAS is associated with high fish mortality [26,27]. It is common to co-isolate *A. hydrophila* and other pathogens from fish suffering from MAS signs, including such pathogens as *E. ictaluri*, *F. columnare*, or *Vibrio parahaemolyticus* [28,29]. There is significant antigenic diversity among *A. hydrophila* strains, with 44 different O-antigen serotypes observed among mesophilic *A. hydrophila* strains [30]. Still, more recent descriptions of *A. hydrophila* strains isolated from diseased fish are not commonly serotyped. The diversity of bacteria within the *A. hydrophila* complex that infect fish [31] and the ubiquitous presence of *A. hydrophila* within aquatic ecosystems, particularly in biofilms and sediments [32], pose significant challenges for the generation of fish vaccines that are broadly protective against *A. hydrophila*.

A significant challenge for fish farmers was the emergence of a hypervirulent pathotype of *A. hydrophila* causing MAS in farmed carp species. This hypervirulent strain was first reported in China in 1989 [33] and later in farmed catfish in the southeastern United States in 2009 [34]. Fish infected with hypervirulent *A. hydrophila* (vAh) experience a rapid onset of disease and very high mortality. This virulent strain was estimated to have caused more than USD 35 million in economic losses since the first appearance in the US industry [9,35].

The development of vaccines to protect farmed fish against *A. hydrophila* was reported beginning in the 1970s. The strategies to vaccinate fish include using bacterins (inactivated cells), live-attenuated bacteria, and recombinant vaccines. *A. hydrophila* bacterins prepared by formalin or heat inactivation were reported to protect channel catfish [36], walking catfish (*Clarias batrachus* L.) [37], goldfish (*Carassius auratus*) [38], rainbow trout (*Oncorhynchus mykiss*) [39], carp (*Cyprinidae*), and loaches (*Misgurnus*) [40]. These bacterins triggered strong adaptive immune responses and were shown to stimulate *A. hydrophila*-specific antibody titers and peroxidase activities in walking catfish [37], goldfish [38], and rainbow trout [39]. Likewise, *A. hydrophila* bacterins were observed to increase the expression of immune-related functions such as IgM, IL-10, and lysozyme in carp and loaches [40].

The use of live-attenuated *A. hydrophila* vaccines can trigger more intense and prolonged immune responses by introducing avirulent bacteria by intraperitoneal injection [41], as was demonstrated in common carp (*Cyprinus carpio* L.) [42] and Indian major carp species (*Catla catla*, *Labeo rohita*, *Cirrhunas mrigala*) [43]. The specific antibody titer in common carp was significantly increased by vaccination with live-attenuated *A. hydrophila* compared to fish vaccinated with formalin-killed vaccine [42]. For Indian carp species, vaccination with a hemolysin-negative *A. hydrophila* mutant induced significant protection (relative percent survival [RPS] >80%) and strong agglutinating antibody response against virulent *A. hydrophila* [43].

Recombinant vaccines were evaluated for their ability to protect rohu (*Labeo rohita*) [44] and American eel (*Anguilla rostrata*) [45] against *A. hydrophila*. In addition, *Escherichia coli* was used to over-express pathogen genes, such as the outer membrane protein gene of *A. hydrophila*, which stimulated IgM levels and lysozyme and significantly reduced fish cumulative mortality rates when challenged by *A. hydrophila* [44,45].

Experimental challenges with vAh strains isolated from US catfish result in significant mortality rapidly, and the vast majority of fish that succumb to disease die within 24 h [46]. The structure of the group 4 capsular polysaccharide and LPS-associated O-antigen from a vAh strain isolated from a US catfish has a novel structure [47] unlike that of other *A. hydrophila* isolates from fish and a capsular polysaccharide-exporting mutant of the well-characterized catfish vAh isolate ML09-119 was observed to be attenuated in its virulence [48]. A catfish-derived vAh strain selected for resistance to two antibiotics demonstrated significant protection in channel catfish and Nile tilapia when IP-injected, resulting in 86–100% protection relative to naïve fish [49]. Attenuated vaccines to protect fish from vAh were generated by selecting for antibiotic resistance [50] or generating attenuated vAh via multiple gene deletions [51]. An attenuated vAh strain resistant to both novobiocin and rifampicin was used to vaccinate channel catfish by IP, resulting in 100% protection against the parent vAh strain with evidence for a strong antibody-mediated response and induction of Na(+)/K(+) ATPase α subunit, hepcidin, interleukin-1β, and lysozyme c within the anterior kidney in vaccinated fish relative to naïve fish [50]. In a study that deleted five vAh genes (*aerA*, *hly*, *ahp*, *alt*, and *ast*) to produce an attenuated mutant strain, researchers elicited a strong adaptive immune response in grass carp (*Ctenopharyngodon idella*). The mutant strain yielded an RPS of 70 or 75% when fish were vaccinated by immersion and subsequently challenged by two different vAh strains. An RPS of 75 or 85% was obtained when fish were vaccinated by intracelomic injection and challenged by vAh strains [51].

Besides using attenuated vAh strains, the extracellular proteins (ECPs) can serve as antigens that elicit a protective response in channel catfish [52,53]. The antiserum from the vaccinated fish agglutinated both vAh cells, and more than 68 pathogenic proteins were recognized and aggregated by catfish IgM, including aerolysin and hemolysin. Furthermore, all channel catfish immunized with vAh ECP (2 micrograms) and Freund’s adjuvant by intraperitoneal (IP) injection survived challenge, whereas naïve fish injected with adjuvant alone all died within five hours [52]. Furthermore, ECP-immunized sera from channel catfish could be used to passively immunize channel catfish and provide an RPS of 85% by two days post-vaccination [53].

The infection of vAh significantly induced transcription of apolipoprotein A1 [54], chicken-type lysozyme [55], G-protein coupled receptor 18 [56], and goose-type lysozyme [57] in kidney, liver, and other tissues of channel catfish. The lysozymes were expressed in the *E. coli* expression system and exhibited high lytic activity against the pathogen. The recombinant vaccines provided 100% protection to catfish two days after IP injection, and the protection remained at 77–100% two to four weeks post-vaccination when challenged by vAh AL09-71 [29,30,31,32]. Similar approaches using recombinant expression of vAh-derived aerolysin, hemolysin [58], ATPase [59], fimbrial proteins [60], immunogenic outer membrane proteins [61], and aerA (hemolytic and cytolytic factor) [62] were used to vaccinate channel catfish, resulting in 58–98% protection relative to naïve fish. Collectively, these studies demonstrate that multiple vaccination strategies can be effective in protecting vAh to farmed fish. Ultimately, the vaccination strategy adopted by fish producers will need to provide long-lasting adaptive immunity and be cost-effective to be widely used. An important question to be addressed by future research is whether any of the vaccines developed to protect fish against vAh strains will provide immunity against other *A. hydrophila* types that are ubiquitous in aquatic ecosystems.

### 2.3. Flavobacterium columnare

*Flavobacterium columnare* is the causative agent of columnaris disease with a worldwide distribution. The disease was first described in the early 1900s in the state of Iowa (USA), in which a thorough investigation of the disease was conducted [63]. Examination of infected tissue under a microscope revealed the responsible bacterium tended to form columns or haystacks; thus, the names *Bacillus columnaris* and columnaris disease were proposed for the bacterium and disease, respectively [63]. *Bacillus columnaris* was first cultured in 1944 [64] on low nutrient media and was reclassified as *Chondrococcus columnaris*. The bacterium was reclassified several times as *Cytophaga columnaris* [65], *Flexibacter columnaris* [66], and finally, as *F. columnare* [67]. Research revealed a striking degree of genetic variation among isolates of *F. columnare*, and phylogenetic analyses defined four distinct genetic groups within the species [68]. These groups not only differ at the genomic level but also in host associations. For example, genetic group 1 isolates are predominantly associated with disease in salmonids, and genetic group 4 isolates are associated with disease in tilapia (*Oreochromis* spp.) aquaculture [68]. Historically, genetic groups 1, 2, and 3 were associated with columnaris diseases cases in the catfish industry [69].

In the US catfish industry, columnaris disease is another leading cause of mortality. In east Mississippi and west Alabama alone, yearly losses attributed to *F. columnare* range between 1.5 and 2.4 million pounds for each region [70] (Bill Hemstreet, Alabama Fish Farming Center, Greensboro, AL USA, personal communication). Mortalities in extreme cases have reached 90%, and in commercial ponds, mortalities reached 50–60% [9], resulting in USD 30 million in economic losses. Columnaris disease has a bimodal distribution. Most cases occur in the spring and fall when pond temperatures change; however, recent increases in cases during the summer months were noted. Clinical signs include gill, skin, and fin necrosis with skin lesions, often with a yellowish color due to the pigmentation of *F. columnare*. Infections may be exclusively external, internal (systemic), or a combination of both [28]. Juvenile catfish are more susceptible to columnaris disease; however, the disease may occur during any phase of commercial production. Diagnosis of columnaris disease is achieved by observation of clinical signs, presence of long slender Gram-negative rods in wet mounts of affected tissues, and isolation of *F. columnare* colonies from tissues characterized as adherent to agar, yellowish in color, and rhizoid colony morphology. Of importance is the common documentation of co-infections upon examination of columnaris disease cases. For example, Hawke and Thune (1992) examined 99 bacterial disease cases in catfish, and greater than 50% represented co-infections [28].

Effective and practical vaccination is highly desired by catfish producers to reduce the impact of columnaris disease. Early research evaluated simple formalin-inactivated bacterins administered by immersion that showed some beneficial effects, including reduced mortality and antibiotic use [71]. Subsequent research resulted in the commercialization of a live-attenuated vaccine that showed good efficacy in the laboratory [72]. Efficacy under production conditions was variable, and the use of the vaccine by the catfish industry declined [73]. As such, several new vaccine platforms were assessed as mitigation tools, with many focusing on the outer membrane proteins (OMPs) as important antigenic regions [74].

Furthermore, a recombinant vaccine (comprised of *F. columnare* chaperone protein DnaK) was evaluated by [75] and showed promise for vaccine efficacy. Similarly, a new live-attenuated vaccine was tested under conditions similar to production. The results demonstrated a beneficial effect, including lower food conversion ratios and larger average weight at harvest [76]. However, there are currently no effective vaccines available in the catfish industry; thus, the prevention of columnaris disease relies heavily upon using good pond management practices to reduce risk factors such as stress, handling, and poor water quality. Epizootics can and will occur with these in place and require treatment using approved antibiotics or other compounds.

**Table 1:** Summary of *E. ictaluri*, *F. columnare*, and virulent *A. hydrophila* infections in catfish culture.

Pathogen	Clinical Signs	Prevalence/Mortality	Antibiotic Therapies ^1^	Vaccination
*E. ictaluri*	Hemorrhaging, exophthalmia, cranial ulcerations (hole-in-head), white nodules in liver tissue, change in swimming behavior, ascites build-up in the abdomen [10]	Up to 47% percent of catfish disease cases in MS and over USD 60 million in losses per year [9,10]	Florfenicol and Sulfadimethoxine–ormetoprim	Live-attenuated developed at Mississippi State University being used in the MS catfish industry [20]
*F. columnare*	Gill, skin, and fin necrosis or lesions and systemic/acute variations [28]	50–60% reported in commercial ponds, but higher mortality (90%) reported [9]. A total of 1.5–2.4 million pounds of losses in East MS and West Alabama [70]	Florfenicol	Recombinant [75] and live-attenuated in development [76] for catfish
Virulent *A. hydrophila*	Hemorrhaging, exophthalmia, reddened fins, ulcerations, abdominal swelling [25,26,27]	Up to USD 35 million in economic losses in the U.S. since 2009 [9,35]	Oxytetracycline dihydrate	Live-attenuated [49,50] and recombinants [58,59,60,61,62] previously reported for catfish

^1^ Based on current U.S. Food and Drug Administration Center for Veterinary Medicine (FDA-CVM) approvals.

## 3. Bacterial Co-Infections

Co-infections are frequently seen in nature and arise when two or more pathogens infect one host. Infections can occur from two primary pathogens infecting the host concurrently, or one pathogen can develop as a secondary infection [77]. Mixed infections also made determining the primary cause of mortality exceedingly difficult, thus increasing treatment difficulties. Though co-infections are so frequent in typical fish environments, the associated information is scarce. Co-infections can exist between bacterial pathogens, viruses, and parasites, allowing for various clinical manifestations and complications for treatment regimens in pond environments [77].

Information on dual bacterial infections is highly limited when compared to that of single bacterial co-infections. Bacterial co-infections are known to cause drastic effects in increasing the extremity of other diseases and grossly increasing mortality, changing host-susceptibility, and duration of infection [77]. Farmers frequently under-report bacterial co-infections leaving little data related to outbreaks, including diagnosis, the immune response of the host [77], and clinical signs. Clinical signs arising from co-infections can be difficult to distinguish due to a lack of information on which pathogen is responsible for which sign of infection. Other infectious agents occurring concurrently with primary pathogens are often characterized as secondary infections or opportunistic, resulting in most research being focused on primary pathogen infections. Co-infections change fish susceptibility to various pathogens [77], resulting in outbreaks causing high mortality. Interactions between the pathogens can lead to bacterial load variability, where loads can be either both suppressed, increased, or one potentially suppressed while the other is increased, although extraordinarily little information is known about how loads are affected during co-infections. Competition between host resources is typical in co-infections; modifying immune activity against other pathogens can suppress or increase the immune response leading co-infections to be either synergistic or antagonistic affecting and altering the host-pathogen interactions [77]. Antagonistic effects allow the primary pathogen to obstruct the secondary pathogen, while synergistic effects create immunosuppressive effects allowing both pathogens to infect the host and increase mortality.

The co-infective ability of bacterial fish pathogens warrants further investigation to better comprehend natural exposure in production systems. In cobia (*Rachycentron canadum*), *Vibrio harveyi*, and *Photobacterium damselae* were used in experimental co-infective challenges. Differences in mortality were observed in fish receiving multiple pathogens compared to some of the single-pathogen treatment groups [78]. In rainbow trout, a co-infective pathogen challenge with novel family *Flavobacteriaceae* isolates also showed increased mortality compared to single-isolate treatments [79]. Similarly, systemic infection and ulcerative dermatitis were observed within farmed barramundi (*Lates calcarifer*), and the cause was attributed to co-infection with *Streptococcus iniae* and *Shewanella algae* [80]. A co-infection of *Yersinia ruckeri* and *Pseudomonas fluorescens* also caused mortality rates of up to 40% across three rainbow trout production farms in Turkey [81]. *Cyprinus carpio* var. koi experienced high mortality rates in Tianjin breeding farms. Moribund koi carps were cultured for bacteria, and *A. veronii* and *V. cholerae* were isolated and identified for the first time in combination by Han et al. (2021) [82]. Both pathogens presented similar clinical signs, including lesions along the liver, intestine, and spleen. Fish also exhibited intestinal hemorrhaging. The research indicated additional studies should be conducted to study pathogenicity further. This work could aid in developing treatment along with future prevention methods [82]. A study conducted by Chandrarathna et al. (2018) examined the effects of co-infection in zebrafish [83]. *A. hydrophila* and *A. veronii* were identified as the causative pathogens inducing mortality amongst zebrafish and were found to be multidrug-resistant. Once challenged, single infections with the pathogens caused less mortality than the co-infections suggesting that mixed infections of *A. hydrophila* and *A. veronii* have higher pathogenicity than single infections [83].

In order to assess the full extent of co-infection outbreaks within catfish production, studies must be conducted to quantify multiple pathogens’ effects on host mortality. *Pangasianodon hypophthalmus* (striped catfish) were observed to have encountered natural infections of *E. ictaluri* in Thailand [84]. In addition, researchers discovered striped catfish were experiencing co-infections of *F. columnare* and *E. ictaluri*. The investigation into the outbreak aimed to characterize both single and dual infections from each pathogen. Clinical signs from *E. ictaluri* and *F. columnare* were consistent between naturally occurring and induced infections, and fish co-infected via immersion challenge displayed higher mortality than catfish injection-challenged single isolates [84]. Thus, researchers fulfilled Koch’s postulates by infecting fish with the recovered isolates and provided molecular markers to better identify outbreaks in fish.

Similarly, striped catfish were immersed in both *E. ictaluri* and *A. hydrophila*, and the results indicated the co-infection caused 95% cumulative mortality. In comparison, the single infection of *E. ictaluri* only had 80% and *A. hydrophila* 10% [85]. Grizzle and Kiryu (1993) also found that channel catfish that displayed latent *A. hydrophila* infection following experimental challenge also exhibited infections with *Acinetobacter* spp., *Plesiomonas* spp., and *Pseudomonas* spp.) [25]. Nofal and Abdel-Latif (2017) also reported a variety of mixed bacterial and bacterial–parasitic infections in African catfish, with the prominent bacterial pathogens *Vibrio* spp., *A. hydrophila*, and *E. tarda* recorded from the pond fish kills [86].

In 2017, researchers at the E. W. Shell Fisheries Center at Auburn University (Auburn, AL, USA) observed chronic mortalities in channel catfish within an in-pond raceway system [87]. Mortalities were deemed unusual due to outbreaks occurring at lower water temperatures and during periods of reduced feeding. After further investigation, three different bacterial pathogens were isolated, indicating a co-infection. Gram-negative bacteria *A. veronii* and *S. putrefaciens* were identified along with the Gram-positive bacterium *S. parauberis*. Fish were exposed to pathogens in an attempt to identify the primary causative agent. Both *A. veronii* and *S. parauberis* were unsuccessful in inducing mortality, while exposure to high doses of *S. putrefaciens* induced signs of disease and low mortality rates (33–50%). Researchers concluded infection with *A. veronii*, *S. parauberis*, and *S. putrefaciens* was a novel co-infection. Future investigations should be conducted to determine the transmission and pathogenicity of *S. parauberis* and *S. putrefaciens* [87]. A summary of the discussed bacterial co-infection reports is presented in Table 2.

Parasites are frequently seen in combination with bacterial pathogens. Researchers investigated whether parasites can act as vectors for bacterial pathogens, increasing infection rates along with mortality. Parasites increase host susceptibility by creating portals of entry for potential bacterial pathogens resulting in high mortality and stress. However, their ability to act as vectors is unknown, and furthermore, the ability of bacterial infections may also potentiate parasitic infections. *Bolbophorus damnificus* is a parasitic trematode responsible for mortalities in commercial ponds in Mississippi. When co-infections between *B. damnificus* and *E. ictaluri* occur, mortality rates were documented to increase dramatically [88]. Thus, *B. damnificus* could potentially create a portal of entry, allowing higher host susceptibility to ESC. Similarly, proliferative gill disease (PGD) could present a portal of entry for bacterial infections through damage and hemorrhaging of the gill in channel catfish. PGD results from a myxozoan parasite, *Henneguya ictaluri*, causing branchial inflammation and the breakdown of chondrocytes [89]. The exposure of hemorrhaged gills due to PGD could allow *A. hydrophila* or other bacterial pathogens a route of transmission into the host’s blood system. This *Aeromonas* spp. infection, in combination with PGD, substantially increases mortality rates, thus increasing economic losses [89]. Simultaneous infections amongst these pathogens facilitate increased exposure to bacterial infections. Similarly, although not reported in catfish species, a combination of *A. hydrophila* and *Epistylus* spp. causing “red sore disease” has also become of recent interest for fish disease diagnostics in freshwater systems [90]. *Ichthyophthirius multifillis* (ich; white spot disease), a freshwater protozoan that causes high mortalities within the industry, was investigated for its potential in acting as a vector for *E. ictaluri*. After investigation, evidence supported ich’s ability to act as a vector, as researchers concluded transmission of bacterial diseases could be increased through parasitic vectors [91]. Yusoff et al. (2020) also reported *A. hydrophila* as a secondary pathogen to *Dactylogyrus* spp., attributing the external, parasite-yielded injuries to the bacterial sites of entry [92]. In addition to these parasitic co-infections, *Aeromonas* spp. were also identified alongside fungal infections in fish species [93,94].

Immune responses associated with co-infections should be further studied to develop future avenues of treatment and prevention [77]. The mucosal surface of catfish is an important immune component to investigate during co-infections. Mucosal surfaces of channel catfish are the first line of defense against pathogens thriving in aquatic environments [95]. Investigating fish mucus’s innate immune defense mechanisms can lead to a better understanding of how pathogens attach and enter the host and aid in developing prevention methods. *F. columnare* is a prime example of a bacterial pathogen that is dependent on attachment to host mucosal surfaces to cause infection. Most studies on this focus primarily on the liver, spleen, and kidney immune factors; however, by examining the expression patterns within the mucus, researchers can determine whether attached bacteria suppress host immune responses [96]. The immune response of the mucosal surfaces of channel catfish was also investigated during *A. hydrophila* infections. Vital lectins and proteins were observed to be altered, potentially enhancing the pathogen’s ability to disrupt and adhere to the mucosal barrier [96]. Though studies were conducted to determine single pathogen effects on the mucosal surface of catfish, similar studies were not conducted to document multiple pathogen effects.

## 4. Diagnostic Summary of Recent Bacterial Co-Infections in Alabama and Mississippi

While diagnostic case records are fraught with submission bias, they can still provide valuable insight into the disease prevalence in the catfish industry. Bacterial diseases are the most commonly diagnosed diseases for catfish case submissions (each case submission is a composite sample of fish collected from a single pond on a given day) at the Alabama Fish Farming Center (AFFC) in Alabama and the Aquatic Research and Diagnostic Laboratory (ARDL) in Mississippi. In addition, Mississippi and Alabama are the top producing catfish states in the US, each having farms that produce channel catfish and hybrid catfish (♀, *Ictalurus punctatus* × ♂, *I. furcatus*). The top prevalent co-infection patterns differ between these states, which may reflect the different system types and the production type, as MS produces more catfish fingerlings than AL.

In 2021, data from the disease databases were mined to summarize the instances of observed co-infections in catfish cases. Analysis of the ARDL data revealed bacterial co-infections occurred more frequently among the two most diagnosed bacterial diseases, ESC and columnaris disease (Table 3). The AFFC records showed that cases of co-infection in Alabama were primarily the bacteria *F. columnare* and either *A. hydrophila* or various other *Aeromonas* spp., including *A. sobria*, *A. caviae*, and *A. veronii*. The next frequently recorded co-infections were between *E. ictaluri* and *F. columnare* (Table 4). These co-infection trends are consistent year-to-year for the AFFC and ARDL from 2016 to 2020.

There are some consistent trends within the Alabama and Mississippi records. Channel catfish represent most of *E. ictaluri*-*F. columnare* co-infection cases and are at least twice, if not more, the number of hybrid catfish cases. Within these cases, it is difficult to assign with certainty which is the primary pathogen as each can cause disease by itself. While *F. columnare* is often thought to be secondary, columnaris disease is usually seen earlier in the year when the cooler temperatures are less conducive for *E. ictaluri* infections and may induce susceptibility in fish up for subsequent co-infections. For *E. piscicida*-columnaris disease co-infections, hybrid catfish represent the majority of cases, but this is not unexpected since hybrid catfish are more susceptible to *E. piscicida* infections [97] but are more resistant to ESC [98] and columnaris disease [99]. However, this combination of bacterial diseases is much less common than *E. ictalurid*–*F. columnare* infections. Therefore, columnaris disease is likely a secondary infection based on the severity of *E. piscicida* lesions compared to the *F. columnare* lesions.

The ARDL data showed co-infections between *F. columnare*–*A. hydrophila* and *F. columnare*–*Aeromonas* spp. infections; the latter of which are cases where the species of *Aeromonas* could not be speciated by the BD BBL^TM^ Crystal^TM^ Enteric/Nonfermentor (Becton Dickinson and Company, Sparks, MD, USA), were significantly lower than those reported in Alabama. With respect to a reduced prevalence of bacterial co-infections discerned throughout the winter months, water temperatures at this time are usually outside of the optimal temperatures for most bacterial pathogens seen in catfish aquaculture. However, there are co-infections involving oomycetes and bacterial pathogens (e.g., *Saprolegnia* spp. and columnaris disease) during this period where the mucus layer becomes thinner due to rapid drops in water temperature [100].

In 2017, there were five *Yersinia ruckeri* cases in hybrid catfish from one farm in Mississippi, one of which was a *Y. ruckeri*-columnaris disease co-infection [L. Khoo, personal communication]. While typically considered a coldwater fish pathogen, *Y. ruckeri* is occasionally seen in warm water fish species, including catfish [101]. No *Y. ruckeri* co-infection cases were diagnosed in Alabama from 2016 to 2020.

## 5. Future Directions for Bacterial Co-Infection Mitigation and Research

A more comprehensive understanding of bacterial co-infections will present many new avenues to promote fish health within catfish production. By further capturing mechanisms for infectivity and virulence and detailing predominant pathogens in diagnostic casework, treatment regimens may be more customized for enhanced efficacy. For instance, correctly identifying primary and secondary pathogens will allow for the appropriate selection of antibiotic or chemical treatment means. As we have limited approved drugs for use in cultured fish species, judiciously administering antibiotics enables producers to retain treatment efficacy. Similarly, as we detail more information on the prevalence and dynamics of antibiotic sensitivity in aquaculture pathogens, the importance of profiling antibiotic susceptibility of multiple pathogen infections is clear. Additionally, if co-infective pathogens’ presence and role are discerned, more rearing-related parameters (i.e., water quality, temperature, feed administration) may be manipulated to cater to the primary effector. Finally, further discerning expanded treatment efficacy has significant economic implications for catfish producers, as treating large ponds for diseases can be very expensive for chemical treatments (aside from medicated feed expenses).

Concerning research directions to explore bacterial pathogens co-infections, access to case diagnostic profiles (both on a small and meta-scale) will provide directions for strain selections that best characterize ongoing health concerns in production ponds. There is a need to establish more natural multi-pathogen in vivo challenge models that best represent the role of both primary and secondary effectors. For instance, dose-concentration studies and timing of pathogen introductions during an in vivo challenge are essential aspects of emulating natural conditions related to disease onset. From this data, different mechanisms of infectivity and changes to pathogenesis during co-infection events can be discerned using molecular tools (i.e., gene expression and sequencing) and growth dynamics.

Furthermore, the cross-protective ability of vaccines used in catfish culture is also crucial to multi-infection mitigation plans. Optimizing the catfish vaccine to provide an expanded umbrella of protection will also potentially reduce bacterial co-infections through an enhanced immune system response and/or shared protective antibodies that are cross-reactive. Finally, aside from chemical treatments and prophylactics, the ability to select genetic lines that are more disease resistant to selected bacterial pathogens would also benefit catfish producers. Several catfish strains and types (i.e., genetic crosses or transgenics) have established evidence for some aspects of disease resistance, yet the expansion and determined scope of these resistance capabilities is of interest.

Co-infective bacterial pathogens in the catfish production sector are not well reported in the literature and warrant further investigation to characterize their pathogenesis in production systems fully. Through the advanced analysis of disease diagnostic data and expanded, targeted research aims, the role of co-infective bacterial pathogens may be further elucidated to control pathogens in catfish aquaculture better.

## Figures and Tables

**Table 2:** Summary of bacterial co-infections discussed in this review.

Fish Species	Pathogens	Co-Infection Mortality	Reference
*Rachycentron canadum*	*Vibrio harveyi/Photobacterium. damselae*	100%	Ramachandra et al. [78]
*Lates calcarifer*	*Streptococcus iniae/Shewanella algae*	~10%	Erfanmanesh et al. [80]
*Oncorhynchus mykiss*	*Flavobacterium* spp./*Chryseobacterium* spp.	36%	Bruce et al. [79]
	*Yersinia ruckeri/Pseudomonas fluorescens*	<40%	Dinçtürk et al. [81]
*Cyprinus carpio*	*Aeromonas veronii/Vibrio cholerae*	50–100%	Han et al. [82]
*Danio rerio*	*Aeromonas hydrophila/Aeromonas veronii*	72.5%	Chandrarathna et al. [83]
*Pangasianodon hypophthalmus*	*Edwardsiella ictaluri/Flavobacterium columnare*	86.7–100%	Dong et al. [84]
	*Edwardsiella ictaluri/Aeromonas hydrophila*	95%	Crumlish et al. [85]
*Ictalurus punctatus*	*Aeromonas hydrophila/Acinetobacter* spp./*Plesiomonas* spp./*Pseudomonas* spp.	N/A	Grizzle and Kiryu. [25]
	*Aeromonas veronii/Shewanella purefaciens/Shewanella parauberis*	80%	Mohammed and Peatman [87]
*Clarias garipenus*	*Vibrio* spp./*Aeromonas hydrophila/Edwardsiella tarda*	N/A	Nofal and Abdel-Latif [86]

**Table 3:** Mississippi State University—College of Veterinary Medicine Aquatic Research and Diagnostic Laboratory, Stoneville; MS Polymicrobial cases from 2016 to 2020. Disease diagnosis as a percentage of total case submissions. CH = Channel catfish, HY = hybrid catfish, BL = blue catfish, OS = Other species.

Disease	Jan	Feb	Mar	Apr	May	Jun	Jul	Aug	Sep	Oct	Nov	Dec	Total	%	CH	HY	BL	OS
***2020 Polymicrobial Diagnostic Cases (N = 763)***																		
*F. columnare*; *A. hydrophila*							1						1	0.13	1			
*F. columnare*; *Aeromonas* sp.						2							2	0.26	2			
*E. piscicida*: *F. columnare*					6	5		2		1			14	1.83	2	12		
*E. ictaluri*; *A. hydrophila*							1						1	0.13	1			
*E. ictaluri*; *F. columnare*			2	11	11	11	4	67	45	7	2		160	20.97	118	42		
*E. ictaluri*; *F. columnare*; *Aeromonas* sp.				1									1	0.13	1			
***2019 Polymicrobial Diagnostic Cases (N = 721)***																		
*F. columnare*, *A. hydrophila*			1										1	0.14	1			
*F. columnare*, *Aeromonas* sp.					2	1							3	0.42		3		
*E. piscicida, F. columnare*					2	2	1		2				7	0.97	1	5	1	
*E. ictaluri*, *F. columnare*					10	8	22	26	27	17			110	15.26	84	26		
***2018 Polymicrobial Diagnostic Cases (N = 660*)**																		
*F. columnare*, *Aeromonas* sp.								1					1	0.2		1		
*E. piscicida*, *F. columnare*					2	1							3	0.5		3		
*E. ictaluri*, *Aeromonas* sp.										1			1	0.2		1		
*E. ictaluri*, *F. columnare*				1	6	2	8	41	14	13	1		86	13.0	70	16		
***2017 Polymicrobial Diagnostic Cases (N = 861)***																		
*F. columnare*, *Aeromonas* sp.			2		1								3	0.3	3			
*E. piscicida, F. columnare*			1	1	1	2		1	4	1			11	1.3	1	10		
*E. ictaluri*, *F. columnare*				1	19	28	16	51	44	25	1	1	186	21.6	155	31		
*E. ictaluri*, *E. piscicida*											1		1	0.1		1		
*Y. ruckeri, F. columnare*			1										1	0.1		1		
***2016 Polymicrobial Diagnostic Cases (N = 744)***																		
*F. columnare*, *Aeromonas* spp.				1			1	1					3	0.4	1	2		
*E. piscicida, F. columnare*				1		1	2	1	3	2	2		12	1.6	1	11		
*E. piscicida*, *F. columnare*, *A. hydrophila*								1					1	0.1		1		
*E. ictaluri*, *A. hydrophila*										1			1	0.1	1			
*E. ictaluri*, *F. columnare*				2	13	10	11	21	28	23	1		109	14.7	79	30		

**Table 4:** Alabama Fish Farming Center polymicrobial cases from 2016 to 2020. Disease diagnosis as a percentage of total case submissions. CH = Channel catfish, HY = hybrid catfish, BL = blue catfish, OS = Other species.

Disease	Jan	Feb	Mar	Apr	May	Jun	Jul	Aug	Sep	Oct	Nov	Dec	Total	%	CH	HY	BL	OS
***2020 Polymicrobial Diagnostic Cases (N = 306)***																		
*F. columnare*; *A. hydrophila*		2	4	2	2	8		2	3	2			25	8.4	22	3		
*F. columnare*; *Aeromonas* sp.			2			5	3		2				12	4	11	1		
*E. piscicida*: *F. columnare*							1						1	0.3		1		
*E. ictaluri*; *A. hydrophila*				1									1	0.3	1			
*E. ictaluri*; *F. columnare*			5		4	3	4	1	1	3			21	7	17	4		
***2019 Polymicrobial Diagnostic Cases (N = 287)***																		
*F. columnare*, *A. hydrophila*				2	7	5	4	5	5				28	9.8	23	5		
*F. columnare*, *Aeromonas* sp.			5	10	1	1		4					21	7.3	14	7		
*E. piscicida, F. columnare*							1						1	0.3		1		
*E. piscicida, Aeromonas spp*							1						1	0.3	1			
*E. ictaluri*, *A. hydrophila*								3					3	1	6			
*E. ictaluri*, *Aeromonas* spp.								10					10	3.5	4	2		5
*E. ictaluri*, *F. columnare*							6	4	1				11	3.8	8	3		
***2018 Polymicrobial Diagnostic Cases (N = 296)***																		
*F. columnare*, *A. hydrophila*			1		2					1	1		5	1.7	3	2		
*F. columnare*, *Aeromonas* sp.			4	10	1	5							20	6.8	10	10		
*E. piscicida*, *F. columnare*				3	2								5	1.7	1	4		
*E. ictaluri*, *A. hydrophila*													0	0				
*E. ictaluri*, *Aeromonas* sp.													0	0				
*E. ictaluri*, *F. columnare*				1	1								2	0.7	2			
*F. columnare*, *Pleisiomonas* spp.						1							1	0.3	1			
***2017 Polymicrobial Diagnostic Cases (N = 352)***																		
*F. columnare*, *A. hydrophila*				1	1	1		1	4				8	2.3	5	3		
*F. columnare*, *Aeromonas* sp.		2	6	2	6								16	4.5	10	6		
*E. piscicida, F. columnare*										1			1	0.3		1		
*E. ictaluri*, *A. hydrophila*													0	0				
*E. ictaluri*, *F. columnare*						1			3	1			5	1.4	5			
***2016 Polymicrobial Diagnostic Cases (N = 460)***																		
*F. columnare*, *A. hydrophila*					1	5		3	3	1			13	2.8	6	7		
*F. columnare*, *Aeromonas* spp.			3	8	4		1			2			18	3.9	16	2		
*E. piscicida, F. columnare*													0	0				
*E. ictaluri*, *A. hydrophila*						1				1			2	0.4	2			
*E. ictaluri*, *F. columnare*			1	1	1	1				11	1		16	3.5	13	3		
*E. ictaluri*, *Aeromonas* spp.						2								0	2			
*Streptococcus* spp., *A. hydrophila*							1							0	1			
*F. columnare*, *Pleisiomonas* spp.					1									0	1			

## Data Availability

The data presented in this review are available upon request from the authors.
